# SIRT6 promotes osteogenic differentiation of mesenchymal stem cells through BMP signaling

**DOI:** 10.1038/s41598-017-10323-z

**Published:** 2017-08-31

**Authors:** Ping Zhang, Yunsong Liu, Yuejun Wang, Min Zhang, Longwei Lv, Xiao Zhang, Yongsheng Zhou

**Affiliations:** 1 Department of Prosthodontics, Peking University School and Hospital of Stomatology, Beijing, 100081 China; 2 National Engineering Lab for Digital and Material Technology of Stomatology, Peking University School and Hospital of Stomatology, Beijing, 100081 China

## Abstract

SIRT6 has been identified as an H3K9 deacetylase and a critical regulator of genome stability, telomere integrity, and metabolic homeostasis. *Sirt6*-deficient mice displayed dramatic phenotypes including profound lymphopenia, loss of subcutaneous fat, lordokyphosis and low bone marrow density. Here, we report that SIRT6 regulates osteogenic differentiation independent of its deacetylase activity *in vitro*. Further mechanistic studies showed that SIRT6 involves the cell fate determination by modulating bone morphogenetic protein (BMP) signaling. Unexpectedly, this modulation depends upon P300/CBP-associated factor (PCAF). In addition, we observed impaired SIRT6 expression in bone marrow mesenchymal stem cells and in bone sections of ovariectomized mice. Taken together, our present study provide new insights into mechanisms of SIRT6-regulated MSC function beyond its H3K9 deacetylase activity.

## Introduction

The Sir2 histone deacetylase was first discovered to regulate genomic stability and aging in budding yeast and Drosophila as a chromatin silencer^1^. Seven sirtuin proteins (SIRT1-7) have been identified that share homology with Sir2 in mammals. Among these mammalian sirtuins, sirtuin 6 (SIRT6) is located in the nucleus and is involved in transcriptional silencing, genome stability, and longevity^2, 3^. *Sirt6*-deficient mice develop normally for the first two weeks, but then undergo several acute degenerative processes, before dying at around one month of age4. SIRT6 is tightly bound to chromatin^4^ and is best characterized as a NAD + -dependent deacetylase of histone H3 lysine 9 (H3K9)^5, 6^ and H3 lysine 56 (H3K56)^7, 8^. SIRT6-mediated deacetylation of telomeric H3K9 is required for efficient association of the Werner syndrome (WRN) protein with telomeric chromatin^5^. Human SIRT6 also deacetylates C-terminal binding protein interacting protein (CtIP) and promotes DNA end resection^9^. In glucose metabolism, SIRT6 functions as a corepressor of the transcription factor Hif1α, suppression of which inhibits glycolysis^10^. SIRT6 also controls hepatic gluconeogenesis through PGC-1α and FoxO1^11, 12^. Recently, the critical role of SIRT6 in stem cell regulation has been determined. *Sirt6*−/− mice exhibit impaired proliferation and differentiation of chondrocytes^13^. SIRT6 regulates the levels of H3K56ac and H3K9ac at the promoters of *Oct4*, *Sox2* and *Nanog*, which in turn control embryonic stem cell (ESC) differentiation through Tet-mediated oxidation of 5-methylcytosine (5mC) into 5-hydroxymethylcytosine (5hmC)^14^. These studies revealed novel roles for SIRT6 in the regulation of differentiation and stem cell function.

Mesenchymal stem cells (MSCs) are multipotent stromal cells capable of self-renewal and capable of multilineage mesenchymal differentiation^15–18^. Multiple signaling pathway was identified to govern the commitment and differentiation of MSCs. BMP signaling was one of the key pathway which has been confirmed to regulate the osteogenic differentiation *in vitro* and bone formation *in vivo* ^19^. BMP2 and BMP4 acted as positive regulators of osteogenic differentiation of MSCs, while BMP3 was reported to promote MSC proliferation^20, 21^. It was also confirmed that deletion of BMP2 and BMP4 impaired osteogenesis and led to skeletogenesis defects^22^. BMPs bind two different BMP receptors BMPR1A and BMPR1B, which leads to phosphorylation of Smad proteins. Phosphorylated Smad proteins activate many target genes such as *RUNX2*, which was identified as the master regulators of osteogenic differentiation. In our previous study, we have confirmed that the H3K9 acetylransferase PCAF was a positive regualtor of BMP signaling pathway. We have clarified that PCAF promotes the expressions of *BMP2*, *BMP4*, *BMPR1B*, and *RUNX2* by increasing H3K9 acetylation on the indicated promoters^22^.

In the present study, we clarified that SIRT6 is a critical molecule during the osteogenic differentiation of MSCs. SIRT6 controls osteogenic commitment through BMP signaling in a P300/CBP-associated factor (PCAF) dependent manner. These findings explored a novel cooperator for SIRT6, namely PCAF, and have discovered a new sirtuin-mediated pathway for the regulation of BMP signaling.

## Results

### SIRT6 is a positive regulator for osteogenic differentiation *in vitro*

**Figure 1:**
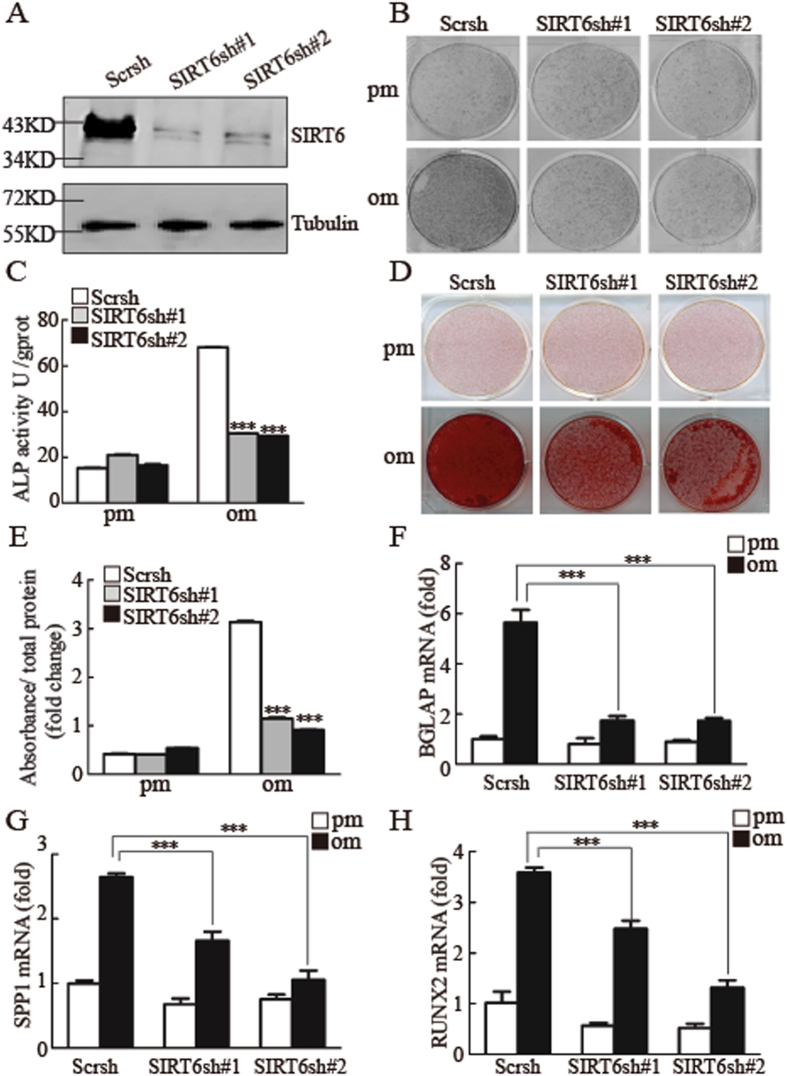
Depletion of *SIRT6* suppresses osteogenic differentiation *in vitro*. (**A**) Western blotting was conducted to examine the knockdown efficiency of SIRT6. (**B**,**C**) *SIRT6* depletion decreased ALP activity as determined by ALP staining (**B**), and ALP quantification assay when cells were cultured in osteogenic media for 7 days (**C**). (**D**,**E**) *SIRT6* depletion impaired mineralization as determined by Alizarin Red S staining (**D**) and quantification (**E**) when cells were treated with osteogenic media for 14 days. (**F**–**H**) SIRT6 knockdown inhibited the expressions of *BGLAP* (**F**), *SPP1* (**G**) and *RUNX2* (**H**), as determined by quantitative real-time RT-PCR. All data are shown as mean ± SD, n = 3. **P < 0.01 and ***P < 0.001. pm: proliferation-inducing media; om: osteogenic-inducing media.

To explore whether SIRT6 was involved in osteogenic differentiation of MSCs, we first constructed *SIRT6* stable knockdown cells with different shRNA sequences. We determined the knockdown efficiency by using fluorescent staining, RT-qPCR and western blotting (SFig. 1A,B & Fig. 1A). Next, both control and *SIRT6* knockdown cells were cultured in osteogenic inducing media for 7 days, then we detected the alkaline phosphatase (ALP) activity by ALP staining and ALP quantification assay. As shown in Fig. 1B,C, SIRT6 knockdown inhibited ALP activity significantly. Moreover, after culturing the cells in osteogenic media for 2 weeks, we conducted alizarin red S staining and quantification assay. As shown in Fig. 1D,E, the extracellular matrix mineralization was impaired in *SIRT6* shRNA treated cells compared with control siRNA treated cells. To further confirm that SIRT6 depletion inhibited osteogenic differentiation in MSCs, we assessed the mRNA expression of several osteogenic markers after induction. As shown in Fig. 1F–H and SFig. 1C, *SIRT6* depletion inhibited the expressions of *BGLAP*, *SPP1*, *RUNX2* and *SP7*. In order to further confirm that the depletion of SIRT6 was specific relative to other SIRTs, we examined expressions of all the sirtuin members in SIRT6 knockdown cell, as shown in SFig. 1D, only *SIRT6* was depleted in SIRT6 knockdown cells, while *SIRT1*, *SIRT2*, *SIRT3*, *SIRT4*, *SIRT5* and *SIRT7* were not affected. Moreover, we detected that SIRT1 knockdown significantly impaired the ALP activity and the expressions of *RUNX2* and *ALP* (SFig. 2A–F), suggested that both SIRT1 and SIRT6 play critical roles in osteogenic differentiation. As a whole, our data suggested that SIRT6 is a positive regulator for osteogenic differentiation *in vitro*.

### SIRT6 regulates osteogenic differentiation independent of its HDAC activity

**Figure 2:**
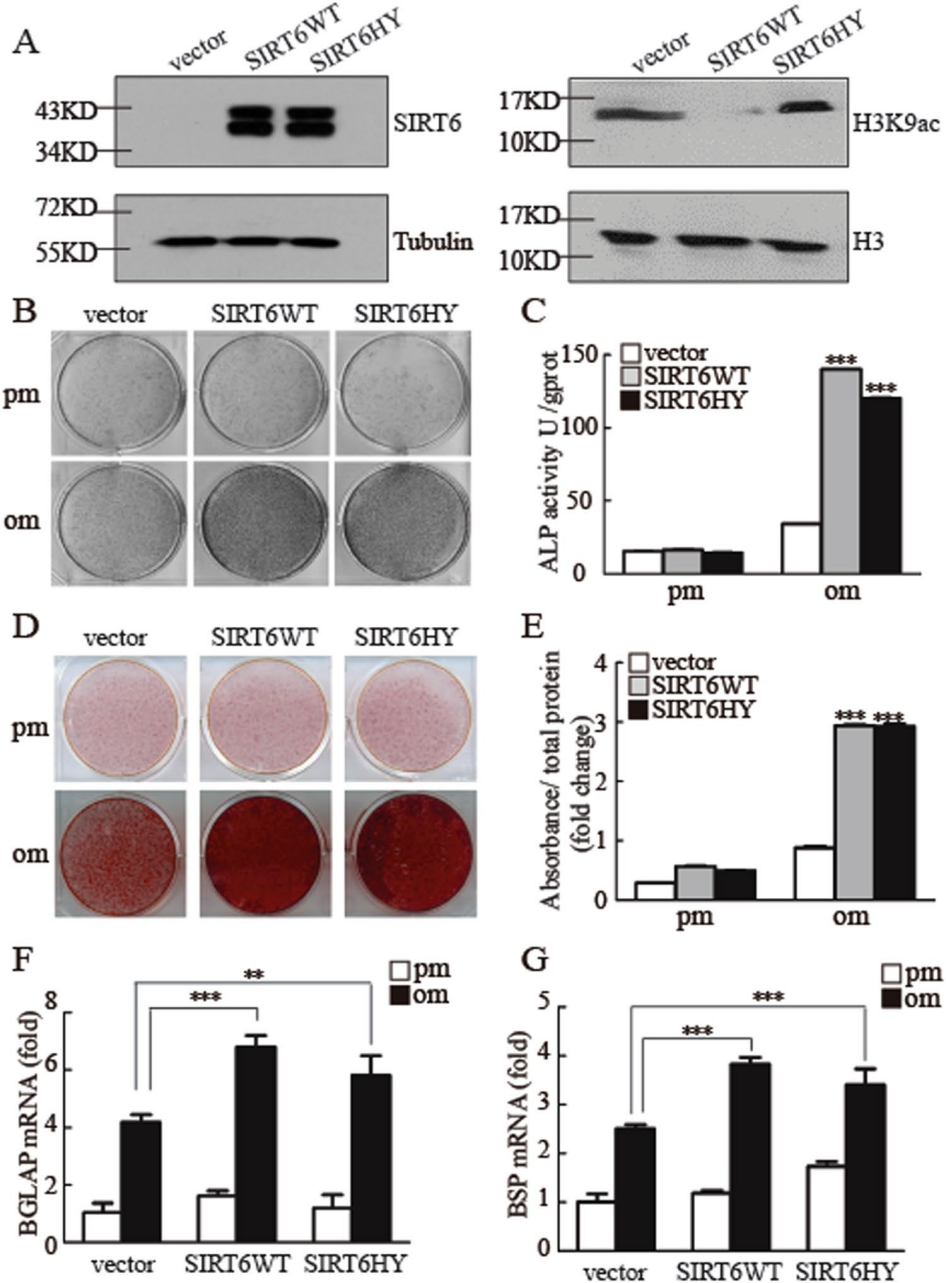
SIRT6 regulates osteogenic differentiation independent of its deacetylase activity. (**A**) Western blotting was conducted to examine the SIRT6 and H3K9ac expression level. (**B,C**) Both WT and mutant *SIRT6* could promote ALP activity by ALP staining (**B**), and quantification assay when cells were treated with osteogenic induction at 7 days (**C**). (**D,E**) Both WT and mutant *SIRT6* could promote mineralization, as determined by Alizarin Red S staining (**D**) and quantification (**E**) when cells were cultured in osteogenic media. (**F,G**) Both WT and mutant *SIRT6* could promote the mRNA expression of *BGLAP* (**F**) and *BSP* (**G**). All data are shown as mean ± SD, n = 3. **P < 0.01 and ***P < 0.001. pm: proliferation-inducing media; om: osteogenic-inducing media.

Elegant studies have showed that the function of SIRT6 is dependent on its deacetylase activity to regulate telomere function, glucose metabolism and chromatin remodeling. To test this requirement, we generated a rescue cell line expressing wild-type SIRT6 or a catalytically dead SIRT6 mutant (SIRT6 H133Y, in which histidine 133 was changed to tyrosine, which disrupts its deacetylase activity) in MSCs/SIRT6 sh, as shown in SFig. 2A,B and Fig. 2A. Unexpectedly, both wild-type SIRT6 and mutant SIRT6 were able to restore osteogenic differentiation, as assessed by ALP staining and quantification (Fig. 2B,C). In addition, overexpression of wild-type and mutant SIRT6 also increased formation of mineralized nodules after treatment with inducing media for 2 weeks (Fig. 2D,E). Consistently, the expression of osteogenic marker genes was rescued in SIRT6 overexpressing MSCs (Fig. 2F–G and SFig. 2B,C). These data suggested that the SIRT6 regulate osteogenic differentiation in a deacetylase activity independent manner.

### SIRT6 regulates osteogenic differentiation through BMP signaling

**Figure 3:**
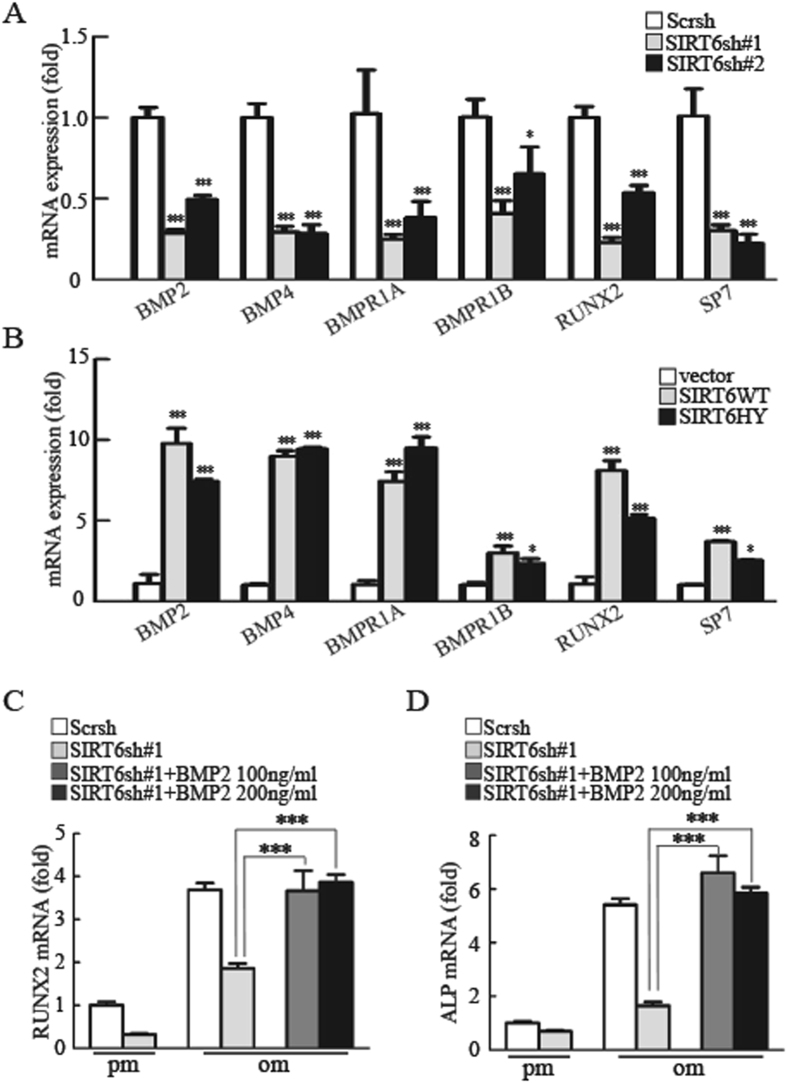
SIRT6 regulates osteogenic differentiation through BMP signaling. (**A**) Real-time PCR analysis of BMP signaling-related genes in WT and *SIRT6* knockdown MSCs. (**B**) Overexpression of WT *SIRT6* and mutant *SIRT6* promoted the mRNA expression of *BMP2*, *BMP4*, *BMPR1A*, *BMPR1B*, *RUNX2* and *SP7*, as determined by quantitative real-time PCR analysis. (**C,D**) RUNX2 (**C**) and Alkaline phosphatase (ALP) (**D**) mRNA expression by quantitative real-time PCR in control, *SIRT6*-Knockdown, *SIRT6*-Knockdown + BMP2 100 ng/ml and *SIRT6*-Knockdown + BMP2 200 ng/ml groups. All data are shown as mean ± SD, n = 3. *P < 0.05, **P < 0.01, and ***P < 0.001.

Several developmental signals have been demonstrated to participate in the differentiation of an osteoblast progenitor to a committed osteoblast, including Hedgehog signaling, Notch signaling, WNT signaling, BMP signaling and FGF signaling^19^. To identify the molecular mechanism underlying the regulation of osteogenic differentiation by SIRT6, we performed quantitative real-time reverse transcription-PCR analysis to examine the expression of key factors in the above-mentioned pathways. We found that the mRNA expressions of *BMP2*, *BMP4*, *BMPR1A*, *BMPR1B*, *RUNX2* and *SP7* were noticeably altered in *SIRT6* knockdown cells (Fig. 3A). In addition, both wild-type and mutant SIRT6 could activate the BMP pathway genes (Fig. 3B). Thus, SIRT6 is a positive regulator of BMP signaling. To further determine the functional connection between SIRT6 and BMP signaling, we next examined whether BMP2 would reverse the osteogenic differentiation in *SIRT6* depletion cells. As shown in Fig. 3C,D and SFig. 3A,B, BMP2, but not the NF-κB inhibitor BAY 11-708, could reverse the reduced *RUNX2* and *ALP* expression caused by SIRT6 deficiency. Taken together, our results indicated that SIRT6 modulates osteogenic differentiation through the BMP signaling pathway, not the NF-κB signaling pathway.

### Regulation of BMP signaling by SIRT6 depends upon PCAF

**Figure 4:**
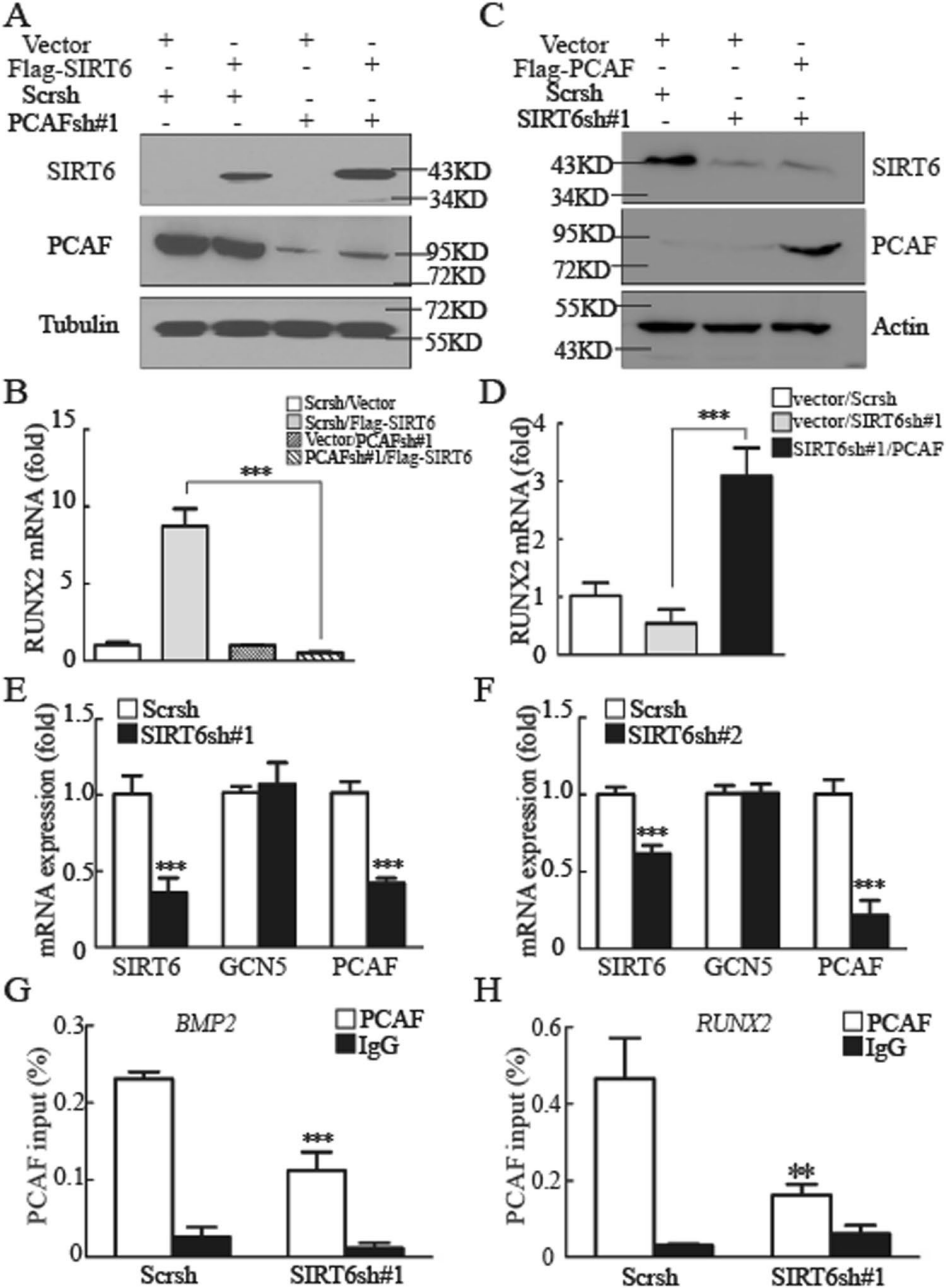
Regulation of BMP signaling by SIRT6 depends upon PCAF. (**A**) Overexpression of Flag-SIRT6 in control and *PCAF*-knockdown MSCs. (**B**) *PCAF* knockdown diminishes the effect of SIRT6 on *RUNX2* expression, as determined by quantitative real-time RT-PCR analysis. (**C**) Overexpression of Flag-PCAF in control and *SIRT6*-knockdown MSCs. (**D**) Overexpression of PCAF in *SIRT6* knockdown cells reversed the decreased expression of *RUNX2*. (**E,F**) The mRNA expression of *PCAF*, but not *GCN5*, was inhibited in *SIRT6* knockdown MSCs. (**G,H**) ChIP analysis detected PCAF at the promoters of *BMP2* (**G**) and *RUNX2* (**H**) in WT and *SIRT6* knockdown MSCs. All data are shown as mean ± SD, n = 3. *P < 0.05, **P < 0.01, and ***P < 0.001.

SIRT6 was recently identified as a specific H3K9 deacetylase that modulates chromatin structure^5, 6^. In the context of gene expression, acetylated H3K9 is associated with actively transcribed genes, whereas H3K9 deacetylation correlates with gene repression^23^. We therefore focused on whether SIRT6′s effects on BMP target genes were being directed through the activities of H3K9 acetyltransferase GCN5 or PCAF. GCN5 was proved to play no effect in BMP pathway in our previous study^24^, while PCAF was confirmed to regulate osteogenic differentiation by activating BMP pathway^22^. As shown in Fig. 4A,B and SFig. 5A,B, although SIRT6 was able to activate the expressions of *BMP2*, *BMP4*, and *RUNX2*, this effect was significantly diminished by eliminating PCAF expression. Additional support for the involvement of PCAF was obtained by overexpression of PCAF. As shown in Fig. 4C,D and SFig. 5C,D, transfection of PCAF rescued the decreased expression of BMP target genes caused by knockdown of *SIRT6*. Thus, SIRT6′s regulation of BMP signaling depends upon PCAF. These data led us to examine the genetic interaction between *SIRT6* and *PCAF* in MSCs. Unexpectedly, we observed significant downregulation of PCAF expression in *SIRT6* depletion cells compared with control cells (Fig. 4E,F). It was confirmed that PCAF binds to *BMP2, BMP4, BMPR1B, and RUNX2* promoters by ChIP assays in our previous work^22^; to further investigate the underlying mechanism, we observed that the knockdown of SIRT6 reduced PCAF binding to the indicated promoters, as shwon in Fig. 4G,H and SFig. 5E–F. Therefore, these data suggested an important function for SIRT6, as a positive modulator of BMP signaling in a PCAF dependent manner.

### SIRT6 was a potential target for osteoporosis treatment

**Figure 5:**
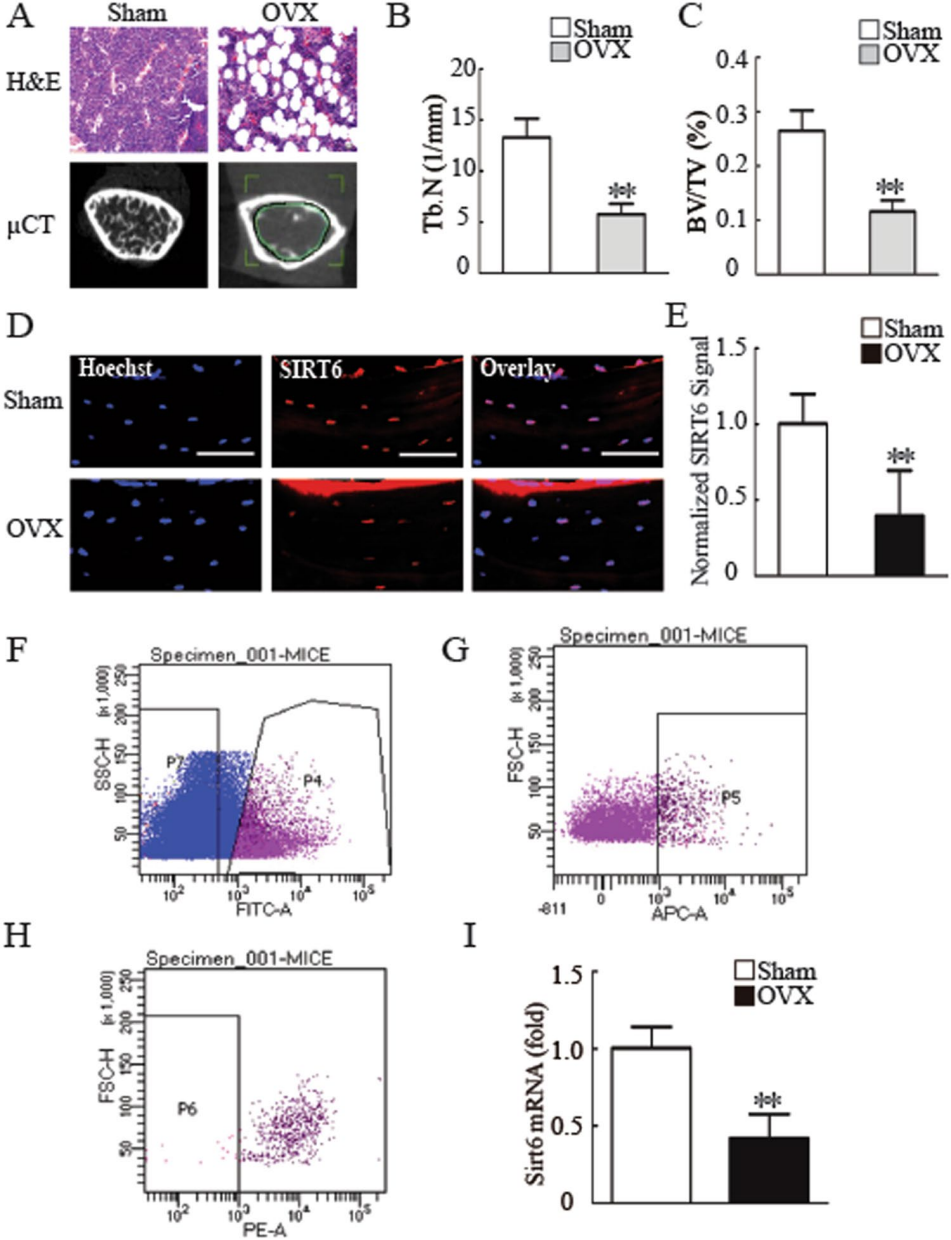
SIRT6 is a potential target for osteoporosis treatment. (**A**) Micro CT and H&E analysis exhibited bone loss in OVX mice compared with sham mice. Scale bar for micro CT and H&E represents 1 mm and 20 µm, respectively. (**B**) OVX mice showed impaired trabecular number compared with sham mice. (**C**) OVX mice showed reduced bone volume compared with sham mice. (**D**) SIRT6 was decreased in bone sections from OVX mice compared with sham mice, as determined by histology and immunofluorescence staining. Scale bar represents 50 µm. (**E**) Quantification of normalized SIRT6 signals in (**D**). (**F**–**H**) Flow cytometry analysis of PDGFR- α^+^Sca-1^+^CD45^−^TER119^−^ cells. (**I**) Compared to sham mice, *Sirt6* expression was dereased in primary MSCs from OVX mice, as determined by RT-qPCR. All data are shown as the mean ± SD, n = 3. **P < 0.01.

It was shown that ovariectomized (OVX) mice were perfect mice model for osteoporosis studying^25^. To verify whether SIRT6 was involved in osteoporosis, we next established OVX mice model. Micro CT and HE assay showed that the OVX mice suffer from severe osteoporosis, as shown in Fig. 5A–C and SFig. 5A,B. We then examined SIRT6 expression in bone sections of both sham and OVX mice by using immunohistochemistry staining. As shown in Fig. 5D,E, the staining of SIRT6 was very weakly in OVX mice compared with sham mice. To further confirm that SIRT6 is a potential target for osteoporosis treatment, we next examined the expression level of SIRT6 in the primary MSCs from the bone marrow of both sham and OVX mice. Platelet-derived growth factor receptor α (PDGFR- α) and stem cell antigen 1 (Sca-1) have recently been identified as selective markers of mouse mesenchymal stem cells (MSCs)^26^. So we isolated a subset of MSCs (PDGFR- α^+^Sca-1^+^CD45^−^TER119^−^) from bone marrow of both sham and OVX mice. As shown in Fig. 5F–H and SFig. 5C, we have obtained a small amount of PDGFR- α^+^Sca-1^+^CD45^−^TER119^−^ cells. To culture these primary mouse MSCs for 1–2 weeks, we collected sufficient MSCs to detect the expression level of Sirt6. As shown in Fig. 5G, compared with Sham mice, the MSCs from OVX mice exhibited decreased Sirt6 expression.

## Discussion

Beyond the recent report of SIRT6′s effect on osteogenic differentiation of rat bone marrow MSCs^27^, our results confirm a clear role for SIRT6 in the regulation of osteogenic commitment of human MSCs, and importantly, establish a previously unidentified underlying mechanism. Sun *et al*., reported that SIRT6 regulates osteogenic differentiation partially via suppressing the nuclear factor-κB signaling pathway^27^. However, in the present study, the impaired osteogenic differentiation triggered by SIRT6 depletion could not be rescued by the addition of an NF-κB inhibitor. We detected that SIRT6 regulates osteogenic differentiation through BMP signaling. The decreased osteogenic differentiation ability was reversed upon treatment with BMP2 in *SIRT6* knockdown cells. In addition, it was reported that SIRT6 deacetylates histone H3 Lysine 9 on promoters of NF-κB target genes to repress NF-κB target gene expression6; nevertheless, we showed that SIRT6 control MSCs’ fate commitment independent of its deacetylase activity. Since the complexity and heterogeneity of MSCs have not been fully reserved until recently, it is reasonable to postulate that SIRT6 may have different functions in rat BMMSCs verses human MSCs.

SIRT6 was considered have no deacetylase activity until 2008, since then, it was shown that SIRT6 play important roles in diverse biological processes through two distinct enzymatic reactions: deacetylation and ADP-ribosylation. For example, SIRT6 directly suppresses the expression of multiple glycolytic genes by interacting with hypoxia inducible factor-1α (HIF1α) and deacetylating H3K9 at the promoters of HIF1α target genes10. Recently, SIRT6 was confirmed to deacetylate H3K56 and interact with SNF2H to accelerate SNF2H translocate to DNA double strand break (DSB) damage sites^28^. In addition, SIRT6 represses the transcription levels of sterol-regulatory element binding protein, SREBP, and those of its target genes, by deacetylating H3K9 and H3K56 at SREBP promoter regions^29, 30^. Acting as a lysine deacetylase, SIRT6 promotes the secretion of tumor necrosis factor-α (TNF-α) by removing the fatty acyl modification on K19 and K20 of TNF-α^31^. SIRT6 also interacts with poly [adenosine diphosphate (ADP)-ribose] polymerase 1 (PARP1) and mono-ADP-ribosylates PARP1, thereby stimulating PARP1 poly-ADP-ribosylase activity and enhancing DSB repair under oxidative stress^32^. It is likely that many of the diverse functions of SIRT6 are carried out via its enzymatic activity. However, SIRT6 is reported to stabilize the DNA-PK catalytic subunit (DNA-PKcs) at chromatin next to an induced site-specific DSB, and it remains unclear whether the enzymatic activity of SIRT6 was required for this function^33^. Interestingly, our study added new evidence that SIRT6 was involved in osteogenic commitment of MSCs, independent of its HDAC activity. To examine whether the effect on osteogenic differentiation depends upon SIRT6′s enzymatic activity, a catalytically incompetent mutant allele of SIRT6 (H133Y) was employed. H133Y had the same effects as WT SIRT6, suggesting that the SIRT6′s effects on osteogenic differentiation is not related to SIRT6′s enzymatic activity. Our data also reemphasized a previously established role for SIRT6 in regulating osteogenic differentiation of rat bone marrow mesenchymal stem cells, albeit in a divergent manner.

To explain the regulation of SIRT6 on BMP signaling, we detected an undiscovered interaction between SIRT6 and PCAF. Our previous study showed that PCAF modulates BMP signaling by increasing histone H3K9 acetylation. In the present study, we detected that SIRT6 regulate osteogenic differentiation via BMP signaling. Interestingly, SIRT6 regulates BMP signaling in a PCAF dependent manner. It is noteworthy that the regulation of PCAF by SIRT6 might extend beyond osteogenic differentiation and have significant implications in undiscovered cellular processes. Thus, further experiments will be needed to identify other targets of PCAF and their effects on cellular physiology.

## Materials and Methods

All of our animal study was approved by the Peking University Biomedical Ethics Committee Experimental Animal Ethics Branch, and were in accordance with the Guide for the Care and Use of the Laboratory Animals (National Academies Press, National Institutes of the Health Publication).

### Isolation and culture of MSCs

We purchased the primary human mesenchymal stromal cells (MSCs) from ScienCell Company (Carlsbad, CA, USA). Cells from three donors were used for the *in vitro* and *in vivo* experiments. To induce osteogenic differentiation, we cultured cells in mineralization-inducing media containing 100 mM/ml ascorbic acid, 2 mM β-glycerophosphate and 10 nM dexamethasone.

### FACS for primary MSCs from mice

First, we collected the femurs and tibias from both sham and OVX mice and crushed the bone with with a pestle. The crushed bones were gently washed once in HBSS + (Hanks-balanced salt solution supplemented with 2%FBS, 10 mM Hepes, and 1% penicillin/streptomucin), then discard the cell suspension after filtered through a cell strainer (Falcon 2350). Then digested the bone fragments for 1 h at 37 °C in 20 ml of DMEM (Invitrogen, Carlsbad, CA, USA) containing 0.2% collagenase (sigma), 10 mM Hepes and 1% P/S. The suspension was filtered with a cell strainer to remove debris and bone fragments, and collected by centrifugation at 280 g for 7 min at 4 °C. The pellet was digested in blood red cell lysing reagent, after which 1 ml of 2* PBS containing 4% FBS (PAA Laboratories GmbH, Linz, Austria) was added, and the suspension was filtered through a cell strainer. Then we stained the cells for 30 min on ice with the follow with the following mABs: APC-conjugated PDGFRα, FITC-conjugated Sca-1, PE-conjugated CD45 and TER119. All mABs were purchased from eBioscience. Flow cytometry analysis and sorting were performed on a FACSCalibur (BD) flow cytometer.

### Retroviral infection and Plasmid Constructions

Viral infection was described before^22^. The target sequences for the shRNA were: *SIRT6sh1*, 5′-AAGCTGGAGCCCAAGGAGGAA-3′; *SIRT6sh2*, 5′-AAGAATGTGCCAAGTGTAAGA-3′.

For gene overexpression, the Flag-SIRT6 and the Flag-SIRT6 HY were cloned into the pLNB vector and then for lentiviral viruses packaging.

### ALP Staining and Quantification

ALP activity was detected by staining with nitroblue tetrazolium and 5-bromo-4-chroro-3-indolyl phosphate. For quantification assay, cells were rinsed twice with phosphate-buffered saline (PBS), trypsinized, and then scraped into ddH_2_O. This was followed by three cycles of freezing and thawing. ALP activity was determined at 405 nm using p-nitrophenyl phosphate (pNPP) as the substrate. A 50 µl sample was mixed with 50 µl of pNPP (1 mg/ml) in 1 M diethanolamine buffer containing 0.5 mM MgCl_2_ (pH 9.8) and incubated at 37 °C for 15 minutes on a bench shaker. The reaction was stopped by the addition of 25 µl of 3 M NaOH/100 µl of reaction mixture. Enzyme activities were quantified by absorbance measurements at 405 nm. Total protein contents were determined using the bicinchoninic acid method using the Pierce (Thermo Fisher Scientific, Rockford, IL USA) protein assay kit in aliquots of the same samples. The samples were read at 562 nm and calculated against a series of bovine serum albumin (BSA) standards. ALP levels were normalized to the total protein content at the end of the experiment.

### Mineralization Assays

The mineralization was determined by staining with alizarin red 14 days after osteogenic differentiation. To quantify matrix mineralization, alizarin red S-stained cultures were incubated in 100 mM cetylpyridinium chloride for 1 hour to solubilize and release calcium-bound alizarin red S into the solution. The absorbance of the released alizarin red S was measured at 562 nm. Data were expressed as units of alizarin red S released per milligram of protein in each culture in a parallel well. This test was repeated three times.

### Acid extraction for histone protein

The cells were suspended in lysis buffer (10 mM HEPES, 1.5 mM MgCl2, 10 mM KCl, 1.5 mM PMSF, 1M DTT, and 98% sulfuric acid) on ice for 30 min, then centrifuge at 11000 g 10 min at 4 °C, keep the supernatant fraction and discard the acid insoluble pellet. Dialyze the supernatant against 200 ml 0.1 M acetic acid twice for 1–2 hours, then dialyze the supernatant against 200 ml H2O for 1 hour 3 hours and overnight, respectively. Finally, the protein can be quantified by using Bio-Rad Protein Assay and the rest are stored at −70 °C.

### Western Blotting

Western blotting was performed as described previously^12^. We obtained the primary antibodies from the following sources: monoclonal antibody to SIRT6 (Cell Signaling, Danvers, MA, USA; Abcam, Cambridge, UK); monoclonal antibody to H3K9 acetylation (Cell Signaling, Danvers, MA, USA); monoclonal antibody to H3 (Cell Signaling, Danvers, MA, USA); monoclonal antibody to PCAF (Cell Signaling, Danvers, MA, USA); monoclonal antibody to α-tubulin (Sigma, St. Louis, MO, USA); and monoclonal antibody to flag (Sigma, St. Louis, MO, USA).

### Quantitative Real-Time PCR

We extracted total RNA using Trizol (Invitrogen) and synthesized 0.5–2 μg aliquots of RNAs using random hexamers and reverse transcriptase (Takara). We performed the quantitative real-time PCR reactions using the QuantiTect SYBR Green PCR kit (Qiagen) and Life Multi-color Real-time PCR Detection System. The primer sequences were as follows: *RUNX2*, (forward) ACTACCAGCCACCGAGACCA, (reverse) ACTGCTTGCAGCCTTAAATGACTCT; *BGLAP*, (forward) CACCATGAGAGCCCTCACACTC, (reverse) CCTGCTTGGACACAAAGGCTGC; *SPP1*, (forward) CAAACGCCGACCAAGGAAAA, (reverse) TGCCTAGGAGGCAAAAGCAA; *BMP2*, (forward) GACTGCGGTCTCCTAAAGGTCG, (reverse) CTGGGGAAGCAGCAACGCTA; *BMP4*, (forward) AGGCCGAAAGCTGTTCACCG, (reverse) TACGGAATGGCTCCATGTTCCC; *BMPR1A*, (forward) TTCCCTGGGGTCCGGACTTA, (reverse) ACGACTCCTCCAAGATGTGGC; *BMPR1B*, (forward)AGCCTGCCATAAGTGAGAAGCAAA, (reverse) GGTGGTGGCATTTACAACGCA; *SP7*, (forward) AACAGGAGTGGAGCTGGCCT, (reverse) GCCATAGTGAACTTCCTCCTGGG; *SIRT6*, (forward) TCAGGCTTCCCCAGGGACAA, (reverse) CATGGTGCCCACGACTGTGT; *PCAF*, (forward) AGGTTCCCCATGGATCTGAAAACC, (reverse) AAAGACTCGCTGTAAGTCTGCCA; *GCN5*, (forward) ATCTGGGCGTCTACCTGGCA, (reverse) ACAGGAATCTGCCATCCCCAGA; Sirt6, (forward) CACAAAACATGACCGCCAGG, (reverse) GCCCCAGATGCTTCATGAGT.

### Micro CT analysis of mice

C57BL/6 mice were obtained from The Jackson Laboratory. Mice were maintained in a pathogen-free facility on a 12 hour light/dark cycle with water and food provided *ad libitum*. Five-month-old mice were sham-operated (sham) or ovariectomized (OVX). After 3 months of ovariectomy surgery, mice were sacrificed for the subsequent assays. The proximal femur and tibia thoroughly dissected free of soft tissue and fixed with 4% paraformaldehyde for 24 h before washing with 10% sucrose solution. Twelve hours later, micro CT of the proximal tibia or femur was performed using a SIEMENS MM Gantry LG CT camera scanner (Germany). Three dimensional images of each proximal tibia or femur were acquired with a voxel size of 10.34 µm in all spatial directions. The samples were scanned at a voltage of 60 KV and a current of 400 µA. A low-pass filter was used to remove noise, and the resulting gray-scale images were segmented. Additionally, an appropriate threshold was adjusted to exactly match the mineralized bone phase. The trabecular parts of the tibia or femur were separated using semi-automatically drawn contours.

### Statistical analysis

All results were performed at least three times. Data were analyzed using the GraphPad scientific software for Windows (San Diego, CA). Comparisons between two groups were analyzed by independent two-tailed Student’s t-tests, and comparisons between more than two groups were analyzed by one-way ANOVA followed by a Tukey’s *post hoc* test. The final results were expressed as the mean ± standard deviation (SD) of 3–10 experiments per group. Values were considered significantly different if P < 0.05.

## Electronic supplementary material

Supplemental Information
