# Supplementary material for: SIRT6 promotes osteogenic differentiation of mesenchymal stem cells through BMP signaling
Source: Sci Rep. 2017 Aug 31;7:10229. doi: 10.1038/s41598-017-10323-z (PMC5578964; doi:10.1038/s41598-017-10323-z)

**Supplemental Information**

# SIRT6 promotes osteogenic differentiation of mesenchymal stem cells through BMP signaling

Ping Zhang[a,b], Yunsong Liu[a,b], Yuejun Wang[a,b], Min Zhang[a,b], Longwei Lv[a,b], Xiao Zhang[a,b], Yongsheng Zhou[a,b,*]

a Department of Prosthodontics, Peking University School and Hospital of Stomatology, Beijing 100081, China

b National Engineering Lab for Digital and Material Technology of Stomatology, Peking University School and Hospital of Stomatology, Beijing 100081, China

* Corresponding author: Yongsheng Zhou, Department of Prosthodontics, Peking University School and Hospital of Stomatology, 22 ZhongguancunSouth Avenue, Haidian District, Beijing 100081, China.

Tel.:+86 10 82195370; fax: +86 10 62173402.

E-mail address: kqzhouysh@hsc.pku.edu.cn

## Supplemental Fig. 1

(A) The knockdown efficiency of *SIRT6* in MSCs was validated by fluorescence microscopy. A1, bright field of Scrsh cells; A2, GFP-Scrsh positive cells; A3, merge of bright field and GFP-positive cells. B1, bright field of SIRT6sh#1 cells; B2, GFP-SIRT6sh#1 positive cells; B3, merge of B1 and B2. C1, bright field of SIRT6sh#2 cells; C2, GFP-SIRT6sh#2 positive cells; C3, merge of C1 and C2. Scale bar represents 100

μm. (B) Knockdown of *SIRT6* in MSCs was validated by RT-qPCR. (C) The knockdown of *SIRT6* inhibited the expression of *SP7* in MSCs, as determined by RT-qPCR. (D) mRNA expressions of SIRT1-SIRT7 in SIRT6 knockdown cells. All data are shown as mean ± SD, n = 3. ****P* < 0.001. pm: proliferation media; om: osteogenic media.

**Supplemental Fig. 2**

(A-B) Knockdown of *SIRT1* in MSCs was validated by western blotting and RT-qPCR. (C-D) *SIRT1* knockdown decreased ALP activity in MSCs. Control or *SIRT1* knockdown MSCs were treated with proliferation- or osteogenesis-inducing media for 7 days for ALP staining (C), and cellular extracts were prepared to quantify ALP activity (D). Knockdown of *SIRT6* inhibited the expression of *RUNX2* (E) and *ALP* (F) in MSCs, as determined by quantitative real-time RT-PCR. All data are shown as mean ± SD, n = 3. ****P*< 0.001. pm: proliferation-inducing media; om: osteogenic-inducing media.

**Supplemental Fig. 3**

(A) Rescue of WT or mutant *SIRT6* cell line, as validated by fluorescence microscopy. A1, bright field of vector-transfection in GFP- SIRT6sh cells; A2, GFP-SIRT6sh positive cells with vector transfection; A3, merge of A1 and A2. B1, bright field of flag-SIRT6 (WT) infection in SIRT6sh cells; B2, GFP-SIRT6sh/ flag-SIRT6 (WT) positive cells; B3, merge of B1 and B2. C1, bright field of flag-SIRT6 (HY) infection in SIRT6sh cells; C2, GFP-SIRT6sh/ flag-SIRT6 (HY) positive cells; C3, merge of C1 and C2.

Scale bar represents 100 µm. (B) Rescue of WT or mutant *SIRT6* cell line was validated by RT-qPCR. (C-D) Overexpression of *SIRT6* promoted the expression of *SP7* (C) and *RUNX2* (D) in MSCs, as determined by RT-qPCR. All data are shown as mean ± SD, n = 3. **P* < 0.05, ***P* < 0.01 and ****P* < 0.001. pm: proliferation media; om: osteogenic media.

**Supplemental Fig. 4**

(A-B) *ALP* (B) and *RUNX2* (C) mRNA expression by RT-qPCR in control, *SIRT6*-Knockdown, *SIRT6*-Knockdown + BAY 117082 (2 µM) and *SIRT6*-Knockdown + BMP2 100ng/ml groups. All data are shown as mean ± SD, n=3. ****P* < 0.001. pm: proliferation media; om: osteogenic media.

**Supplemental Fig. 5**

(A-B) *PCAF* knockdown diminishes the effect of *SIRT6* on *BMP4* (A) and *BMP2* (B) expression, as determined by RT-qPCR analysis. (C-D) Overexpression of *PCAF* in *SIRT6* knockdown cells reversed the decreased expression of *BMP4* (C) *and BMP2* (D). (E-F) ChIP analysis detected PCAF at the promoters of *BMP4* (E) and *BMPR1B* (F) in WT and *SIRT6* knockdown MSCs. All data are shown as mean ± SD, n=3. **P* < 0.05 and ****P* < 0.001.

**Supplemental Fig. 6**

(A-B) Trabecular separation (A) and thickness (B) were detected in both sham and

OVX-ed mice. *$P < 0.05$ and **$P < 0.01$.

**SFig. 1**

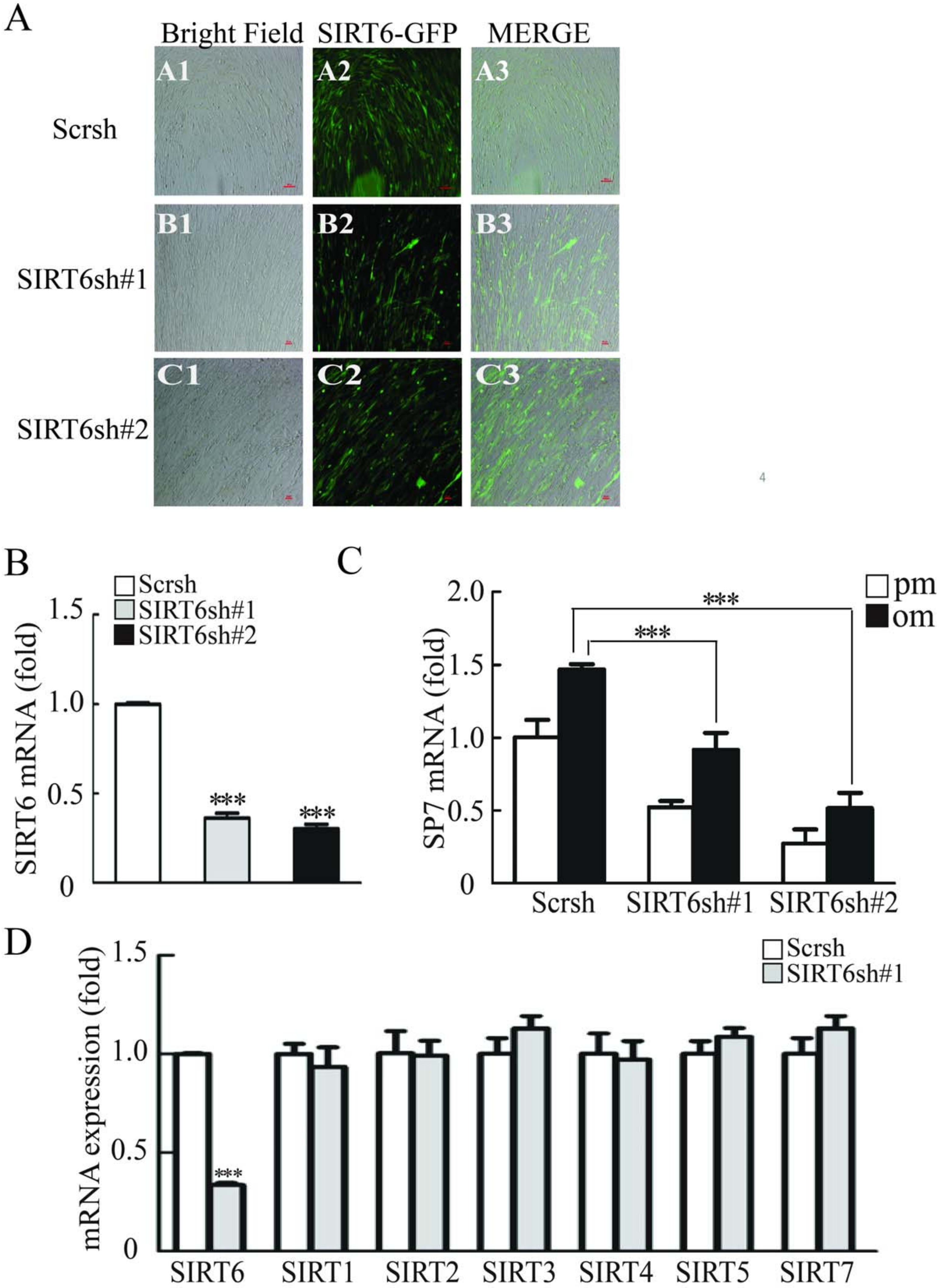
A
Bright Field
SIRT6-GFP
MERGE
A1
A2
A3
Scrsh
B1
B2
B3
SIRT6sh#1
C1
C2
C3
SIRT6sh#2
B
Scrsh
SIRT6sh#1
SIRT6sh#2
SIRT6 mRNA (fold)
1.5
1.0
0.5
0
***
***
C
pm
om
SP7 mRNA (fold)
2.0
1.5
1.0
0.5
0
***
***
Scrsh
SIRT6sh#1
SIRT6sh#2
D
Scrsh
SIRT6sh#1
mRNA expression (fold)
1.5
1.0
0.5
0
***
SIRT6
SIRT1
SIRT2
SIRT3
SIRT4
SIRT5
SIRT7

SFig. 2

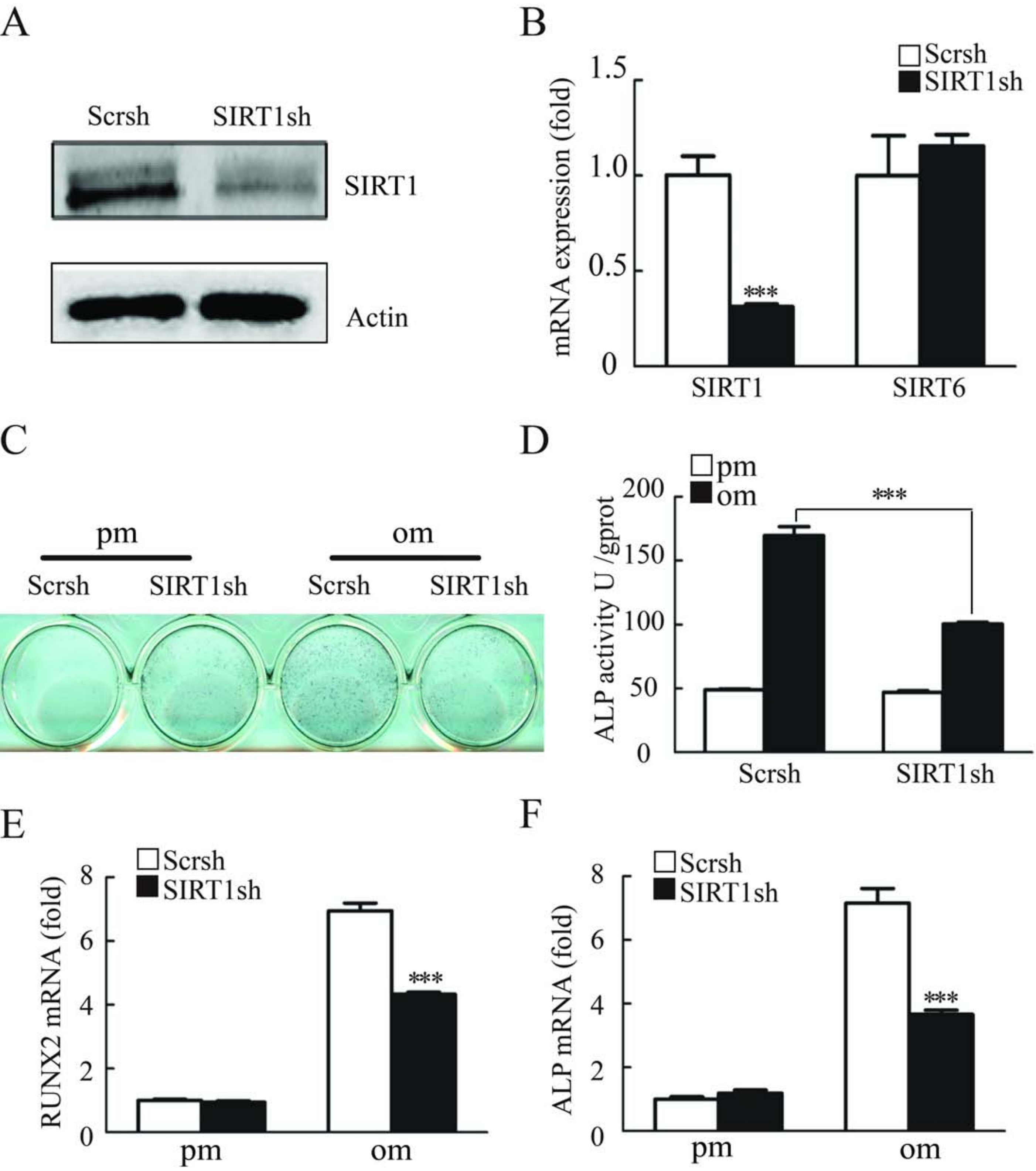
A
Scrsh
SIRT1sh
SIRT1
Actin
B
Scrsh
SIRT1sh
mRNA expression (fold)
1.5
1.0
0.5
0
***
SIRT1
SIRT6
C
pm
om
Scrsh
SIRT1sh
Scrsh
SIRT1sh
D
pm
om
ALP activity U /gprot
200
150
100
50
0
***
Scrsh
SIRT1sh
E
Scrsh
SIRT1sh
RUNX2 mRNA (fold)
8
6
4
2
0
***
pm
om
F
Scrsh
SIRT1sh
ALP mRNA (fold)
8
6
4
2
0
***
pm
om

# SFig. 3

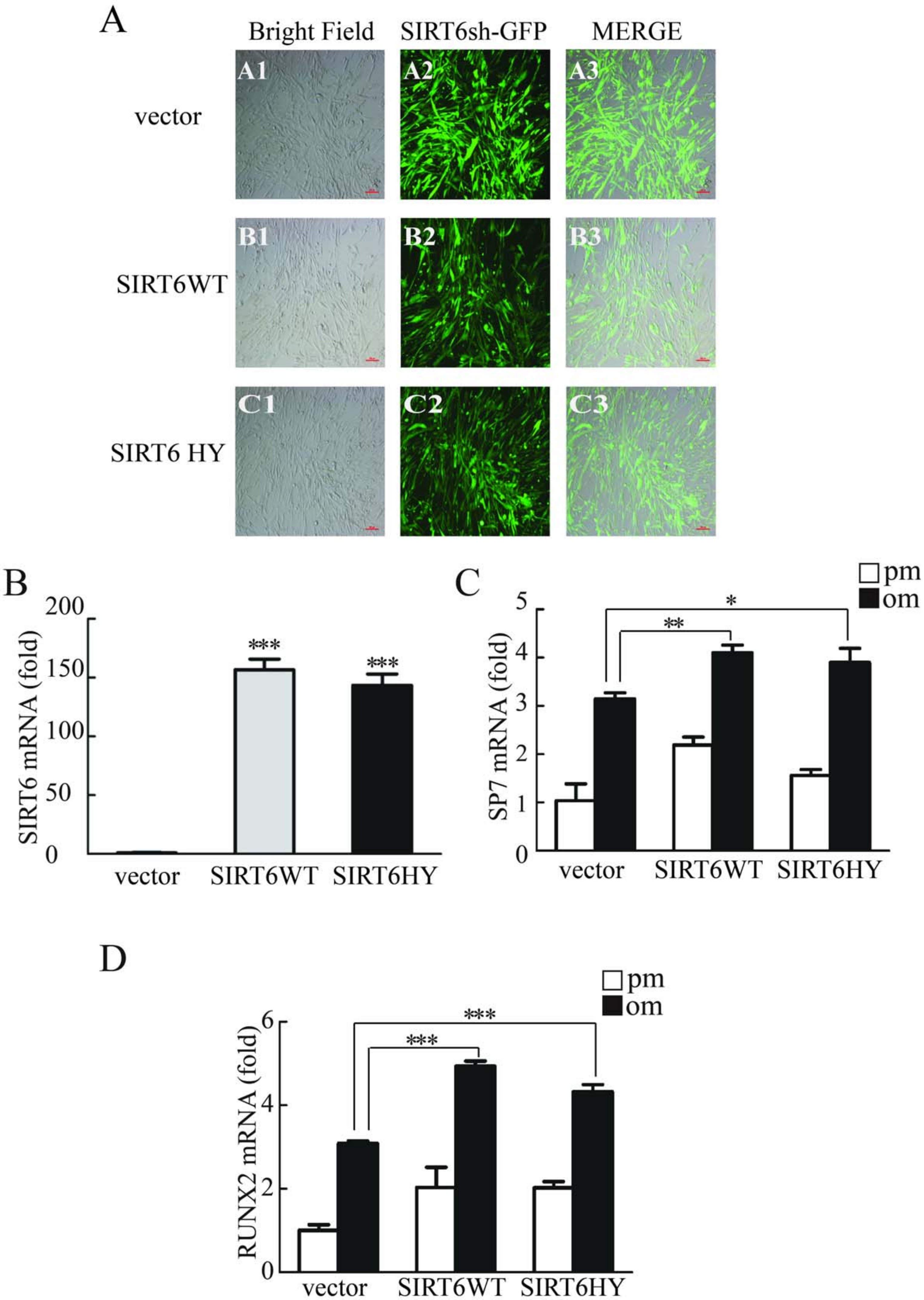
A
Bright Field
SIRT6sh-GFP
MERGE
A1
A2
A3
vector
B1
B2
B3
SIRT6WT
C1
C2
C3
SIRT6 HY
B
200
150
100
50
0
SIRT6 mRNA (fold)
***
***
vector
SIRT6WT
SIRT6HY
C
pm
om
5
4
3
2
1
0
SP7 mRNA (fold)
*
**
vector
SIRT6WT
SIRT6HY
D
pm
om
6
4
2
0
RUNX2 mRNA (fold)
***
***
vector
SIRT6WT
SIRT6HY

## SFig. 4

A

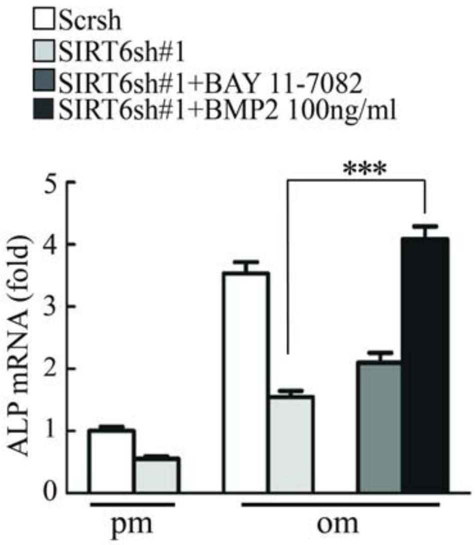
Scrsh
SIRT6sh#1
SIRT6sh#1+BAY 11-7082
SIRT6sh#1+BMP2 100ng/ml
***
ALP mRNA (fold)
0
1
2
3
4
5
pm
om

B

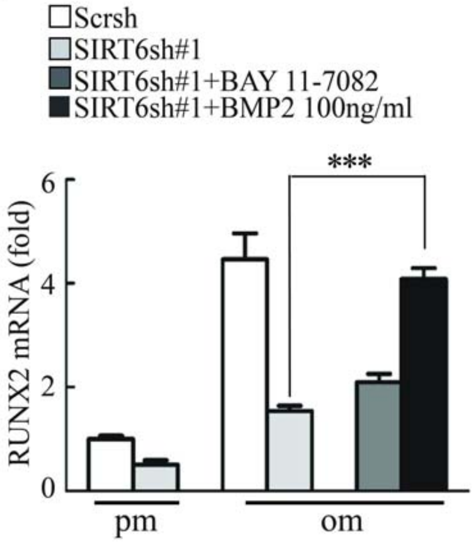
Scrsh
SIRT6sh#1
SIRT6sh#1+BAY 11-7082
SIRT6sh#1+BMP2 100ng/ml
***
RUNX2 mRNA (fold)
0
2
4
6
pm
om

**SFig. 5**

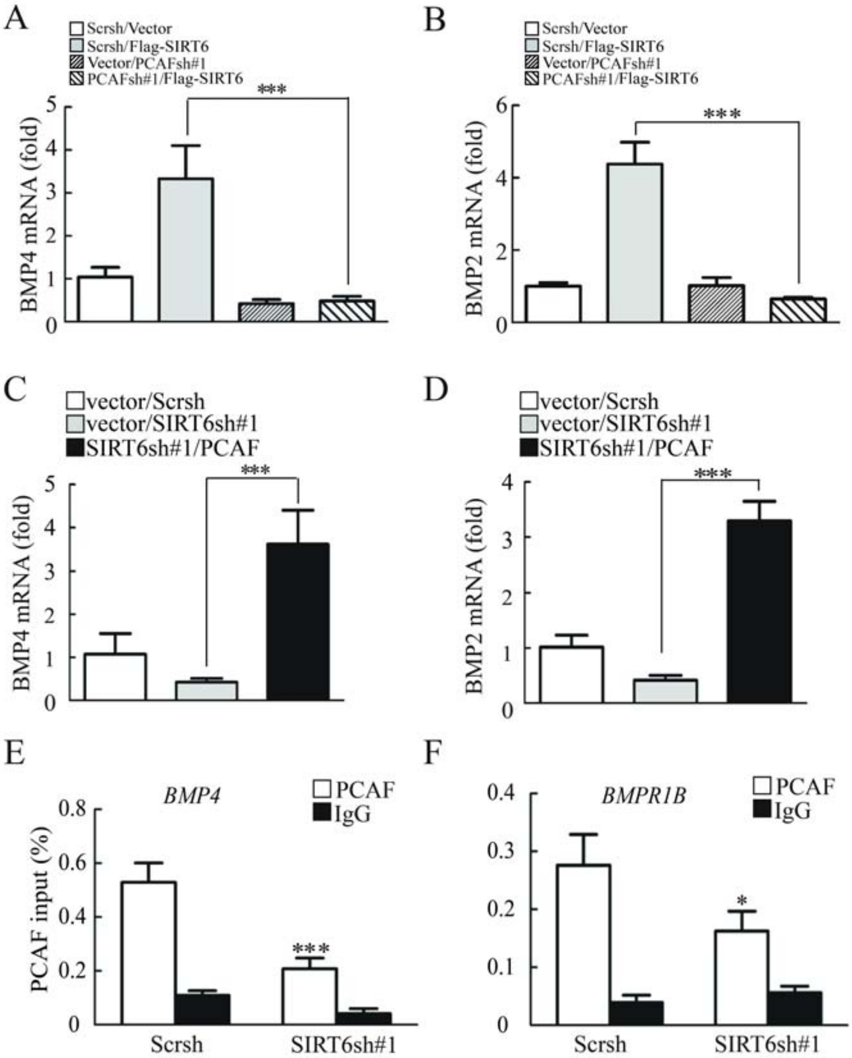
A
Scrsh/Vector
Scrsh/Flag-SIRT6
Vector/PCAFsh#1
PCAFsh#1/Flag-SIRT6
BMP4 mRNA (fold)
***
B
BMP2 mRNA (fold)
***
C
vector/Scrsh
vector/SIRT6sh#1
SIRT6sh#1/PCAF
BMP4 mRNA (fold)
***
D
BMP2 mRNA (fold)
***
E
BMP4
PCAF
IgG
PCAF input (%)
***
Scrsh
SIRT6sh#1
F
BMPR1B
*
Scrsh
SIRT6sh#1

# SFig. 6

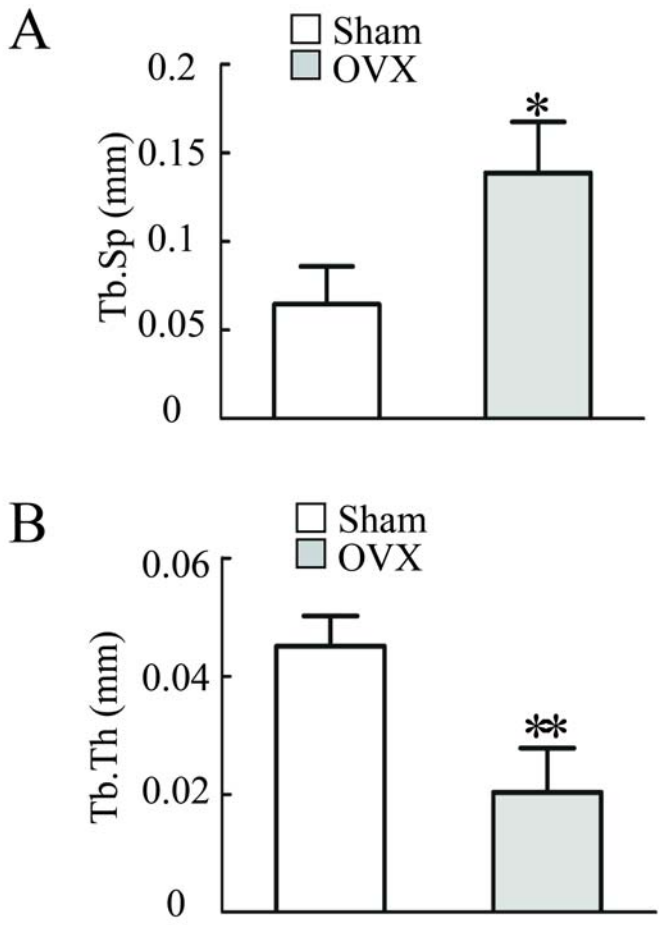

Supplement: Supplementary file 1 — Supplemental Information [file 41598_2017_10323_MOESM1_ESM.pdf]
